# 

**Published:** 1862-08

**Authors:** 


### CONTENTS.

### ORIGINAL CONTRIBUTIONS.

ART. XXIV. Clinical Remarks on the Bowel-Affections of Children in the Medical Wards of the Mercy Hospital. By N. S. Davis, M.D., Professor of Clinical Medicine, etc. ........ 459
ART. XXV. Surgical Notes from the Army. By Henry W. Davis, M.D., ........ 471

### ARMY CORRESPONDENCE.

Letter from E. Andrews, M.D., ........ 479

### SELECTIONS.

The Chlorine and Milk Treatment of Scarlet Fever and the Typhoid Fevers. By Conway T. Edwards, M.R.C.S.E., ........ 485
Epilepsy. After the French of Herpin. By Edward Sutton Smith, M.D., ........ 491
On the Treatment of Suspended Animation Under the Influence of Chloroform. By William Marcet, M.D., F.R.S., Assistant-Physician to the Westminster Hospital, etc., etc., ........ 497
On Local Anæsthesia from Cold, and its Applicability to the Severer Operations. By James Arnott, M.D., ........ 502
On the Pathological Anatomy of Puerperal Fever. By Professor Buhl, 506
Strychnia and Morphia, ........ 508
Health of the British Army and Navy Compared Together, ........ 509

### BOOK NOTICES.

Health: Its Friends and its Foes. By R. D. Mussey, M.D., LL.D., etc. 510
A Practical Treatise on the Diseases of the Throat and Great Vessels, Including the Principles of Physical Diagnosis. By Walter Hayle Walshe, M.D., Fellow of the Royal College of Physicians, etc., etc., 511
Causes and Cure of Diseases of the Feet: With Practical Suggestions as to their Clothing. By C. H. Cleveland, M.D., ........ 512
Communications of the Rhode Island Medical Society for the year 1862. 512

### EDITORIAL.

Chicago Medical Society.—Ovariotomy.—Diseases at Camp Douglas. Camp Dysentery, etc., ........ 512
Prospects of Our Medical Schools, ........ 517
Treatment of Epilepsy, ........ 518
Observations on the Effect of the Preparations of Iron. Translated from the German by D. S. Gans, M.D., Cincinnati, Ohio, ........ 518
Ovariotomy in Paris, ........ 520
Perchloride of Iron and Ingrowing Toe-nail, ........ 521
Ozone Corrective of Miasmata, ........ 521
Cure of Vericose Veins, ........ 521

